# Normal myocardial perfusion values on high-resolution pixel-wise perfusion maps

**DOI:** 10.1186/1532-429X-15-S1-P21

**Published:** 2013-01-30

**Authors:** Amedeo Chiribiri, Geraint Morton, Andreas Schuster, Eva Sammut, Gilion Hautvast, Marcel Breeuwer, Niloufar Zarinabad, Eike Nagel

**Affiliations:** 1 King's College, London, UK; 2 Philips Innovation Group - Healthcare Incubators, Eindhoven, the Netherlands; 3 Philips Healthcare, Imaging Systems, Best, the Netherlands; 4 Eindhoven University of Technology, Biomedical Engineering, Eindhoven, the Netherlands

## Background

The feasibility and pre-clinical validation of pixel-wise quantification of myocardial perfusion on standard and high-resolution first-pass perfusion CMR images has been recently described. However, the diagnostic criteria for the detection of CAD have not yet been defined and normal values in healthy subject are unknown.

Aim of this study was to define normal reference values of stress and rest myocardial perfusion on high-resolution pixel-wise perfusion maps.

## Methods

Ten healthy volunteers (5 males, age 22±5 years, no significant past medical history) underwent high-resolution 3T perfusion (Philips Achieva TX, Best, the Netherlands). Hyperaemia was induced with adenosine administered at 140 mcg/kg/min for 4.5 min. Perfusion imaging commenced 3 min into the infusion and continued for 1.5 min. A dual bolus of weight adjusted of gadolinium contrast agent (Gadobutrol/Gadovist, Bayer, Germany) was injected at 4 ml/s by a power injector (Spectris Solaris EP, MEDRAD, Inc., USA). The dual bolus method was designed to overcome signal saturation effects in the LV while maintaining adequate myocardial contrast to noise. For this study, the dual bolus consisted of equal volumes of 0.0045 mmol/kg followed by 0.045 mmol/kg of the contrast agent, each flushed with 25 ml of normal saline and separated by a 25 s pause. Participants performed a short breath hold during injection of the first (dilute) bolus and a second breath hold for as long as possible during the main bolus of the contrast agent. Quantification was performed using the Fermi deconvolution method.

## Results

**Figure 1:**
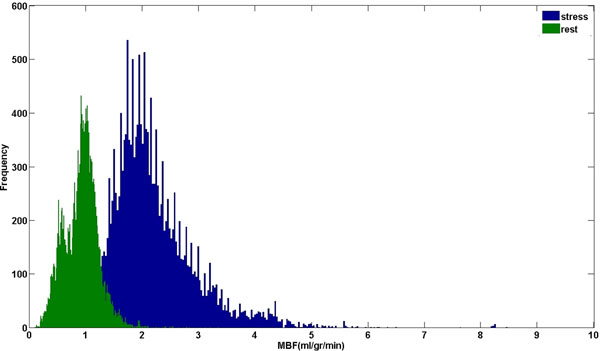
Histogram representing the distribution of stress and rest perfusion values on high-resolution perfusion maps in a population of 10 normal subjects.

Global pixel-wise stress and rest perfusion were 2.3±1.4 ml/min/g and 0.9±0.3 ml/min/g, respectively. The average pixel-wise myocardial perfusion reserve (MPR) was 2.5±0.7. The coefficient of variation (CV) was 0.61 and 0.33 for global stress and rest perfusion, and 0.28 for MPR. The skewness test confirmed the normal distribution of stress and rest perfusion values (1.8 and 1.03, respectively; Figure 1).

These values are relatively close to normal values previously reported for segmental analysis (2.5±0.5 ml/g/min and 0.6±0.1 ml/g/min; Morton et al, Eur Heart J CV Imaging 2012), with the difference of a higher standard deviation. This difference is most likely due to the different levels of noise in the source data and to physiological dyshomogeneities of normal myocardial perfusion throughout the myocardium, averaged out on segmental analysis and maintained on high-resolution pixel-wise assessment.

## Conclusions

Quantitative high-resolution pixel-wise perfusion quantification is a promising technique to assess the presence, the extent and the severity of coronary artery disease. The availability of normal reference perfusion values will enable the definition of criteria of abnormality.

## Funding

The authors acknowledge financial support from the Department of Health via the National Institute for Health Research (NIHR) comprehensive Biomedical Research Centre award to Guy's & St Thomas' NHS Foundation Trust in partnership with King's College London and King's College Hospital NHS Foundation Trust. The Centre of Excellence in Medical Engineering funded by the Wellcome Trust and EPSRC under grant number WT 088641/Z/09/Z. Funded by the British Heart Foundation award RE/08/003.

